# A Compend of Diseases of Children

**Published:** 1891-04

**Authors:** 


A COMPEND OF DISEASES OF CHILDREN. By Marcus P. Hulfield' A. M., M. D.

This is No. 14 of Blakiston, Son & Co.'s series of Query-Compends, that have become so deservingly popular with the medical student. The volume, as the author states in his preface, is founded upon a translation of Dr. Ernest Kormanis, "Compendium der Kinderkrakheiten."

The book is not intended to take the place of larger text-books on pediatrics, but for a ready aid to the student. The divisions and sub-divisions of the different subjects makes it a book that is of more than the ordinary value of query compend.

